# Performance Evaluation of Enzyme Breaker for Fracturing Applications under Simulated Reservoir Conditions

**DOI:** 10.3390/molecules26113133

**Published:** 2021-05-24

**Authors:** Yuling Meng, Fei Zhao, Xianwei Jin, Yun Feng, Gangzheng Sun, Junzhang Lin, Baolei Jia, Piwu Li

**Affiliations:** 1 Key Laboratory of Shandong Microbial Engineering, Shandong Academy of Sciences, Qilu University of Technology, Jinan 250353, China; 1043118305@stu.qlu.edu.cn (Y.M.); 1043119623@stu.qlu.edu.cn (F.Z.); crazyelton@163.com (X.J.); baoleijia@cau.ac.kr (B.J.); 2 Research Institute of Petroleum Engineering and Technology, Shengli Oilfield Company, Sinopec, Dongying 257029, China; fengyun866.slyt@sinopec.com (Y.F.); sungangzheng.slyt@sinopec.com (G.S.); linjunzhang.slyt@sinopec.com (J.L.); 3 State Key Laboratory of Biobased Material and Green Papermaking (LBMP), Shandong Academy of Sciences, Qilu University of Technology, Jinan 250353, China

**Keywords:** hydraulic fracturing, gel breaking, enzyme breaker, mannanase

## Abstract

Fracturing fluids are being increasingly used for viscosity development and proppant transport during hydraulic fracturing operations. Furthermore, the breaker is an important additive in fracturing fluid to extensively degrade the polymer mass after fracturing operations, thereby maximizing fracture conductivity and minimizing residual damaging materials. In this study, the efficacy of different enzyme breakers was examined in alkaline and medium-temperature reservoirs. The parameters considered were the effect of the breaker on shear resistance performance and sand-suspending performance of the fracturing fluid, its damage to the reservoir after gel breaking, and its gel-breaking efficiency. The experimental results verified that mannanase II is an enzyme breaker with excellent gel-breaking performance at medium temperatures and alkaline conditions. In addition, mannanase II did not adversely affect the shear resistance performance and sand-suspending performance of the fracturing fluid during hydraulic fracturing. For the same gel-breaking result, the concentration of mannanase II used was only one fifth of other enzyme breakers (e.g., mannanase I, galactosidase, and amylase). Moreover, the amount of residue and the particle size of the residues generated were also significantly lower than those of the ammonium persulfate breaker. Finally, we also examined the viscosity-reducing capability of mannanase II under a wide range of temperatures (104–158 °F) and pH values (7–8.5) to recommend its best-use concentrations under different fracturing conditions. The mannanase has potential for applications in low-permeability oilfield development and to maximize long-term productivity from unconventional oilwells.

## 1. Introduction

Owing to global reductions in the number of recoverable conventional oil reservoirs, by 2035, oil resources recovered from low-permeability reservoirs will account for approximately half of all newly developed oil production projects. Hydraulic fracturing has proved to be one of the most economically competitive technologies that can be applied to improve productivity and maximize recovery in low-permeability reservoirs [1]. The principle underlying hydraulic fracturing involves the use of high-viscosity fluids to generate high-conductivity fractures and transport proppants into the fractures. Subsequently, high-viscosity fluids should be rapidly converted into a low-viscosity fluid, which should then flow back to the reservoir surface [2]. Therefore, in this study, an efficient and useful enzyme breaker was selected and applied to complete the viscosity reduction process after the fracturing operation to ensure easy flow-back and efficient clean-up of the propped fractures.

In general, the additives to hydraulic fracturing fluids are composed of a thickening agent, a crosslinker, breakers, a proppant, a pH adjustment system, a fungicide, and a clay stabilizer. The most important additives that influence hydraulic fracturing fluids are the thickening agent and breaker. In particular, guar-based polymers are currently the most reliable thickening agents for hydraulic fracturing [3]. Considering their satisfactory viscosity and rheology, they can deliver higher proppant concentrations than their lower-viscosity analogues. In addition, guar gum is also a relatively low-cost natural material with a high affinity for water [4]. Guar-based polymers are high-molecular-weight biopolymers, in which the main chain consists of mannose, with galactose as the side chains, such that the ratio of mannose to galactose varies from 1.6:1 to 1.8:1 [5]. Previous studies have reported that the polymer viscosity, which reflects its stability, exhibits a positive correlation with molecular weight. In guar-based polymers, the β-1,4-glycosidic bond of the mannose main chains is crucial to maintain polymer stability [6,7]. In addition, the α-1,6-glycosidic bonds in the side chain can also experience degradation, thereby reducing the viscosity of guar-based polymers, which is useful for viscosity reduction after fracturing [2]. This feature of guar-based polymers can be used to select suitable breakers that ensure the elimination or minimization of residual gel damage while optimizing well stimulation.

Ideally, to reduce the gel residue, an efficient and adaptive breaker should cleave polymers into low-molecular-weight fragments. Breakers for crosslinked gels are usually categorized as enzymes and oxidizers [8]. Oxidative breakers (e.g., peroxydisulfates, bromates, and peroxides) are most commonly used for the clean-up treatment of residues [9]. However, when they are used in the process of polymer degradation, the process is random and uncontrollable. This is usually because, after fracture formation, oxidizers degrade the guar fluid and reduce the viscosity of the fracturing fluid by producing free radicals owing to thermal decomposition [10,11,12]. Fuller [2,13] estimated that the efficacy of the oxidative breaker is strongly dependent on its activity and concentration. In a lower temperature reservoir, lower oxidant reactivities result in limited thermal decomposition, which may lead to fracturing failure; therefore, the oxidant is difficult to use for the purpose of breaking the gel during the low-temperature hydraulic fracturing process [11]. Therefore, a higher concentration breaker is required in medium-low temperature reservoirs, relative to high-temperature reservoirs. Sarwar et al. [14,15,16] examined the amount of residue remaining after the addition of oxidative breakers to the fracturing fluid over a wide range of temperatures (68–212 °F). In particular, the filter cake formed as a result of the generation of a substantial amount of residue can cause formation fracture blockage and a reduced oil yield. In addition, these oxidizers are often strongly incompatible with many common fracturing additives, such as organic materials, and require special consideration during their deployment and storage [17]. Although they are relatively common and readily available, their low reactivity and uneven residue removal ability do not favor their application in medium-temperature reservoirs.

To overcome these drawbacks, enzyme breakers, which offer the possibility of efficient and controllable degradation while achieving complete gel breakage, have been introduced [18]. In general, the enzymes used are proteins, consisting of amino acid chains, which can degrade polysaccharide polymers via the hydrolysis of the connective bonds; each enzyme degrades a certain chemical bond in the polymer backbone. As biocatalysts for polymer-breaking reactions, enzyme breakers offer significant advantages over traditional oxidative breakers [13]. First, enzyme breakers only hydrolyze the polymers in the hydraulic fracturing fluids, thereby avoiding the occurrence of undesirable reactions that affect the wellbore, formation, or fracturing equipment [17]. Second, they can effectively “break” guar polymers into monosaccharides or oligosaccharides, leading to a significant reduction in the amount of residue generated [19]. Third, the gel-breaking reaction performed using enzyme breakers is mild and controllable, guaranteeing a sufficient supporting force at the initial stage of fracturing construction to enable crack formation and deliver the proppant [20,21]. However, the stability (spatial structure) of enzyme breakers is strongly affected by environmental factors, such as temperature and pH [22]. In general, as the temperature and pH increase, there is an increase in the tendency of the three-dimensional conformation of the enzyme to change from the folded active state to the unfolded inactive state [23]. Therefore, a new breaker is required to break the traditional pH and temperature limits, which can operate under alkaline and medium-temperature reservoir conditions, as well as playing an important role in the field of hydraulic fracturing.

Considering the specificity of enzymes in targeting polymer links and their stabilities with respect to different environmental conditions, previous studies have investigated several high-performance hydrolase breakers that can specifically degrade the glycosidic bonds of guar-based polymers, such as mannanase, cellulase, galactosidase, and amylase [24,25,26,27,28]. However, the existing literature does not include reports on enzyme breakers with higher gel-breaking efficiency in medium-temperature and alkaline reservoirs. Therefore, our research was conducted under simulated medium and low temperatures and alkaline reservoir conditions, to identify the enzyme breaker with the optimum gel-breaking effect under this condition.

Mannanase is a type of enzyme that specifically decomposes β-1,4-glycosidic bonds. Thus far, it has been widely applied in the feed, medicine, and food industries. Although mannanase had not been widely applied in hydraulic fracturing, some progress has been made since its introduction. When compared with other breakers, mannanase can degrade polymers more thoroughly and generates reduced gel residue. Moreover, some authors reported that a certain type of mannanase exhibits higher activity for high temperatures and a wide pH range [29,30,31]. These properties make it a superior enzyme breaker that can be an excellent alternative to the use of harsh treatments in alkaline and medium-temperature formations. However, the effectiveness of this enzyme has not been verified in the site experiments of fracturing operations, and the best-use concentration of the enzyme breaker under different conditions has not been determined. The best amount of breaker can not only achieve the goal of low damage and low cost for the fracturing fluid system, but also achieve the effect of increasing the flowback rate and increasing production of low-permeability reservoirs. Therefore, in this study, the indoor evaluation of the gel-breaking effect and applicability of mannanase and other enzyme breakers was carried out by simulating the reservoir conditions (158 °F, pH = 8.5) of the Xinjiang Shengli Oilfield [32,33,34]. In addition, the best-use concentration of the optimal enzyme breaker has been determined under other broader conditions.

## 2. Materials and Methods

### 2.1. Materials

This study was conducted in 2019 at the Laboratory of Biobased Material & Green Papermaking, Qilu University of Technology, China. The ammonium persulfate breaker (APS), thickening agent, clay stabilizer, fungicide, and other chemical additives were provided by the SinoPEC Shengli Oil Field Ltd. Co. (Dongying, China). A series of enzyme breakers was provided by the Shandong Longda Bio-Products Co., Ltd. (Linyi, China). The crosslinker and pH adjustment system were purchased from the Damao Chemical Reagent Factory (Tianjin, China). The basic components of the fracturing fluid used in this study are listed below.

Thickening agent: A guar gum blend was used as the thickening agent in all of the experiments in this study. The blend consisted of a high viscosity, chemically modified polysaccharide that disperses readily and subsequently self-hydrates to yield a viscous solution.

Crosslinker (0.35% *w*/*w*): Sodium borate was used as a source of borate ions to crosslink the guar gum polymer. The borate ions generated 1:10 complexes with the guar chains.

pH adjustment system: Sodium hydroxide (0.1 M) and hydrochloric acid (0.1 M) were used as the pH adjust system, which was used to maintain optimal pH conditions for the reaction between the thickening agent and crosslinker.

Additives: The clay stabilizer (0.2% *w*/*w*), fungicide (0.2% *w*/*w*), and other chemical additives were added as per their actual use in hydraulic fracturing treatments in the field.

Breakers: Mannanase I (CAS: 37288-54-3), mannanase II (CAS: 37288-54-3), amylase (CAS: 9000-92-4), cellulase (CAS: 9012-54-8), pectinase (CAS: 9032-75-1), xylanase (CAS: 9025-57-4), galactosidase (CAS: 9031-11-2), glucanase (CAS: 9025-70-1), and ammonium peroxydisulfate (CAS: 7727-54-0) were the breakers used in the experiments.

### 2.2. Methods

#### 2.2.1. Effect of Breaker on Shear Resistance Performance and Sand-Suspending Performance of the Fracturing Fluid

In general, guar concentrations in the range of 0.12–0.96% *w*/*w* are used for hydraulic fracturing operations [35]. However, a polymer loading of 0.4% *w*/*w* is more suitable for common oilfield fracturing conditions. Therefore, in our experiments, a guar gum gel concentration of 0.4% *w*/*w* was used. First, a sufficient amount of the guar gum was used to prepare a 500-mL 0.4% *w*/*w* solution. The solution was stirred at 600 rpm for 5 min, additionally, the polymer was allowed to hydrate for 4 h at 200 rpm. Second, 1-mL concentrations of 0.2% *w*/*w* clay stabilizer, fungicide, and other fracture additives were separately added to 50-mL of 0.35% *w*/*w* borax solution. Thereafter, the borax solution and guar polymer were mixed for crosslinking, and the polymer was adjusted to a pH of 8.5. Third, once set up 8 groups of parallel experiments, the enzyme breakers were added separately to 500-mL the cross-linking solution (0.1 g enzyme breaker per liter of solution). The enzyme breakers included mannanase I, mannanase II, amylase, cellulase, pectinase, xylanase, galactosidase, and glucanase [36,37,38]. Finally, the mixed solution was stirred continuously at a rate of 170 s^−1^ and at 158 °F [39]. The viscosity of the fracturing fluids was measured at 20 min intervals using a rotational viscometer to study the effect of different enzyme breakers on the temperature and shear resistance of the fracturing fluid.

Proppant sedimentation experiments were performed with gravel suspended in 0.4% *w*/*w* polymer solution according to a ratio of 3:7 at 158 °F. This experiment evaluates the sand suspending performance of the fracturing fluid with the addition of different enzyme breakers [40,41].

#### 2.2.2. Gel-Breaking Efficiency of Breakers

Based on the temperature and shear resistances of the fracturing fluids, as well as the sand-suspending performance after the addition of the different enzyme breaker [39], the relatively suitable mannanase I, mannanase II, and galactosidase breakers were selected for further experiments. First, the gels were prepared as per the procedure outlined in the previous section [42]. Once set up 3 groups of parallel experiments to conduct the assay, mannanase I, mannanase II, and galactosidase were added separately to a 500-mL mixture of fracturing fluid (0.02 g enzyme breaker per liter) [43,44]. Finally, the change in the viscosity of the guar polymer was measured using a rotary viscometer to study the gel-breaking efficiencies of the different enzymes. The optimal gel breaker based on the breaking efficiency/performance under simulated the reservoir conditions (158 °F, pH = 8.5) of the Xinjiang Shengli Oilfield was selected. We simultaneously conducted comparative experiments to determine the breaking efficiency of the ammonium persulfate breaker.

#### 2.2.3. Reservoir Damage by the Breaker after the Fracturing Operation

Crosslinked guar samples were prepared. The samples were adjusted to a pH of 8.5 and either ammonium persulfate or mannanase II breakers were added to conduct the gel-breaking experiments at 158 °F and other wider temperature range. The samples were left overnight at the breaking temperature to facilitate maximum breakage. The next day, to estimate the amount of unbroken polymer and residue generated after breakage, these samples were subjected to a residue-after-break procedure [45]. The contents were centrifuged at 3000 rpm for 30 min and filtered. To calculate the amount of residue recovered, the gel residue was weighed using weighing paper. Subsequently, the weighting paper and the residue were dried for another 12 h and reweighed to ensure moisture removal.

To further examine the molecular size of the guar polymer after it is “completely broken”. The changes in the molecular size of the guar polymer before and after the gel-breaking under simulated the reservoir conditions (158 °F, pH = 8.5) was analyzed by the laser particle size analyzer [46].

#### 2.2.4. Best-Use Concentrations of Mannanase II under Different Environmental Conditions

In a typical experiment, viscometric assays of guar polymer solutions were performed within a temperature range of 104–158 °F and pH values (7–8.5) conditions with the use of a rotational viscometer, thereby further evaluating the gel-breaking performance of mannanase II and optimal dosage under different conditions [36]. The gels were prepared as per the procedure outlined in Section 2.2.1. The temperature was adjusted by means of water-bath heating while the alkalinity was adjusted by adding the pH regulator. Finally, a rotational viscometer was used to measure the apparent viscosity of the fracturing fluid sample under different temperature and pH conditions to determine optimal enzyme dosage.

#### 2.2.5. Statistical Analysis

Statistical analyses were performed using SPSS v26.0 (IBM, Chicago, IL, USA). One-way ANOVA was performed followed by Duncan test [47], where *p* < 0.05 was considered significant.

## 3. Results and Discussion

### 3.1. Effect of Breaker on Shear Resistance Performance and Sand-Suspending Performance of the Fracturing Fluid

Considering that the breaker and other fracturing additives are simultaneously injected into the well in the fracturing operating process, the fracturing fluid requires a high viscosity to effectively transfer pressure and carry the proppants. Therefore, after adding the enzyme breaker during the fracturing operation, we must consider the effects of the temperature, shear resistance, and suspended sand performance on the fracturing fluids [23].

**Figure 1:**
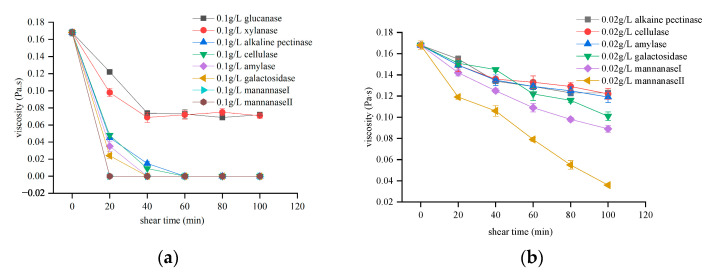
Temperature tolerance and shearing resistance of the fracturing fluid after the addition of different enzyme breakers at a shear rate of 170 s^ࢤ1^ and at 158 °F. (**a**) Comparison of the temperature and shearing resistance performance of the fracturing fluid after the addition of the selected enzymes and (**b**) comparison of the temperature and shearing resistance performance of the fracturing fluid after the addition of the preferred enzymes at low concentrations.

In this study, eight candidate enzymes were selected, their temperature and shear resistance performance with respect to the guar polymers were investigated by analyzing the change in the viscosity of the solution at a shear rate of 170 s^ࢤ1^ and at 158 °F for the addition of each breaker. As shown in Figure 1, all curves first decrease with the shear time and then tend to maintain a plateau when the temperature remains at 158 °F. In Figure 1a, 0.1 g/L concentrations of xylanase and glucanase were unable to maintain a continuous downward trend for the fracturing fluid viscosity. In contrast, the same concentrations of mannanase I, mannanase II, amylase, cellulase, galactosidase, and alkaline pectinase directly caused the fracturing fluid to liquefy, such that the fracturing fluid was unable to complete the task of generating high-conductivity fractures and transporting the proppant into the fractures. Therefore, to reduce the speed of degradation, lower concentrations of these preferred breakers were considered. However, based on related literature and previous experiment judgment, low-concentration cellulase, amylase and pectinase alone cannot completely reduce the viscosity of guar gum polymer [1,5,18]. Comprehensive consideration of cost and degradation speed, lower concentrations of mannanase I, mannanase II, and galactosidase breakers were used. Figure 1b shows that lower-concentration enzymes, particularly mannanase II, can still reduce the viscosity of the guar polymers. Moreover, after shearing for 60 min at 158 °F, the viscosity of the fracturing fluids is greater than 50 mPa·s, satisfying the application requirements for the fracturing fluid [13].

**Figure 2:**
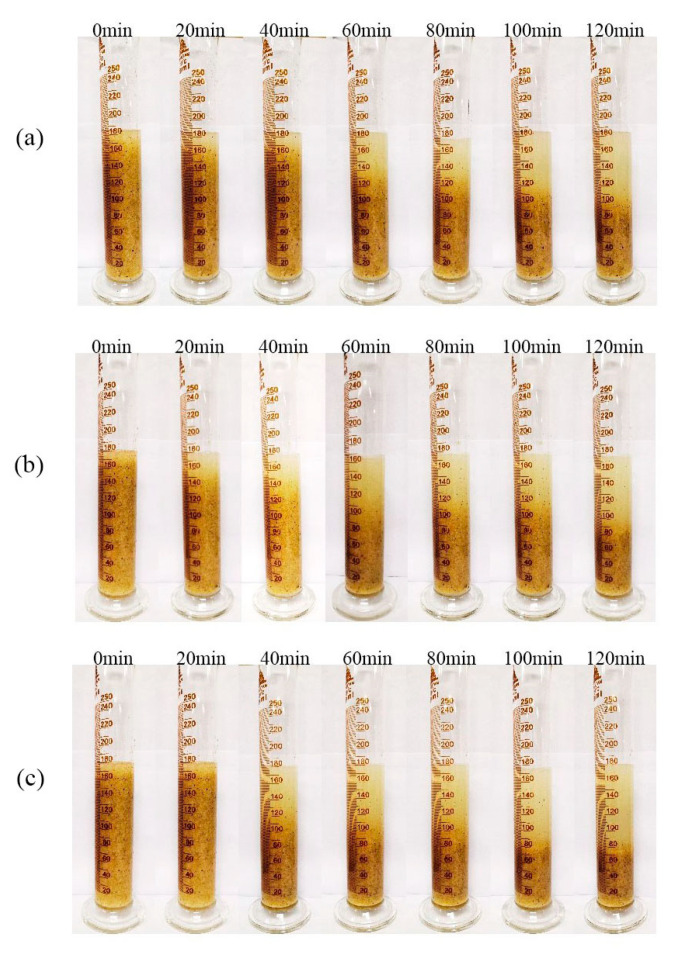
Sand-carrying ability of the fracturing fluid after the addition of the preferred enzymes: (**a**) galactosidase, (**b**) mannanase I, and (**c**) mannanase II.

Fracturing fluids should have high viscosity to carry the proppant in the initial phase; otherwise, low viscosities significantly affect the effectiveness of fracturing [11]. The temperature and shear resistance of the fracturing fluid after adding the preferred enzymes was confirmed. Even if the viscosity of the fracturing fluid after the addition of the mannanase I, mannanase II, and galactosidase declines more rapidly, a high viscosity can be maintained at 158 °F. Therefore, based on our judgment, the fluid also has good sand suspending performance. The proppant sedimentation experiments were performed with 550–830 μm gravel suspended in a guar polymer solution at 158 °F to evaluate the sand suspending performance of the fracturing fluids. Figure 2 lists the state of the suspended sand in the fracturing fluid after adding the preferred enzymes at 158 °F over time. Based on Figure 2, the sand-suspended fracturing fluid after adding mannanase II was still able to carry sand for more than 120 min, which can be ascribed to the excellent viscoelasticity of the fracturing fluid at 158 °F. In comparison, the previous literature also pointed out that the fracturing fluid added with ammonium persulfate breaker may also lose its sand-carrying capacity within 60 min due to excessive degradation [40]. Therefore, these enzymes should have major implications for hydraulic fracturing, such as for further gel-breaking performance research.

### 3.2. Gel-Breaking Efficiency of Breakers

**Figure 3:**
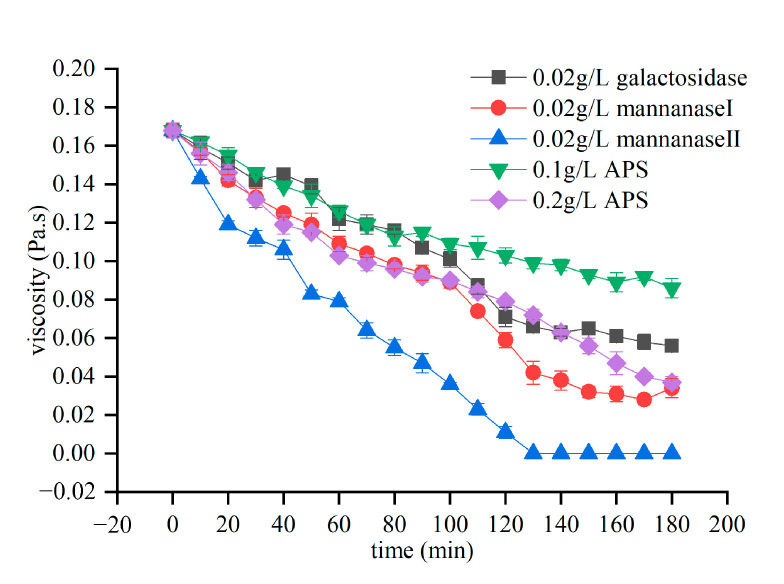
Viscosity changes in the fracturing fluid after adding high-performance breakers under simulated reservoir conditions.

When the fracturing fluid completes the task of carrying the proppant to support new fractures, its viscosity must drop rapidly and turn it into a fluid with strong flowability, facilitating its flow-back to the surface [37,38]. Therefore, we must investigate the gel-breaking efficiency of the breaker. Figure 3 shows the results for the rheology study of the fracturing fluid corresponding to different breakers at 158 °F and a pH of 8.5. The effectiveness of the enzyme breakers can be indirectly measured via viscosity experiments. These results indicate that mannanase II offers significant advantages over other single hydrolases. For a given concentration of all the breakers, mannanase II effectively induced the breakage of the crosslinked polymer, whereas the other enzyme breakers were not as effective. Mannanase II is the key enzyme in the hydrolysis of mannan and randomly degrades the β-1,4-glycosidic linked backbone of mannan [48,49]. The mannanase II is produced by a strain of Bacillus amylose isolated from the konjac field, and after purification, the enzyme shows maximum activity at pH 7.0 and 104 °F with guar gum as substrate and perform high stability at a range of pH 5–9. In, addition, the alkali stability of mannanase II is better, which is slightly higher than the reported enzyme [31,48]. Russell showed that the surface of the protein has a strong protective effect on the catalytic region of the core, and that the alkaline stability of the enzyme is not determined by the electrostatic force of the active center of the enzyme, but by the interaction of multiple factors [50,51]. The stronger alkali stability of mannanase II may be that its surface protects the core catalytic region better, and it is a long process to explain the alkaliphilicity of the enzyme.

Previous studies have shown that oxidative gel breakers can effectively break gels in high-temperature reservoirs, but when they are below the activation temperature, the efficiency of breaking gel will be greatly reduced [10,12,13]. Compared with the ammonium persulfate breaker, an oxidative breaker, mannanase II was highly reactive, such that the overall process afforded a greater reduction in the viscosity. Overall, under simulated Xinjiang Shengli Oilfield reservoir conditions (158 °F, pH = 8.5), mannanase II showed superior performance and in smaller amounts compared with other common breakers.

### 3.3. Reservoir Damage by the Breaker after the Fracturing Operation

**Figure 4:**
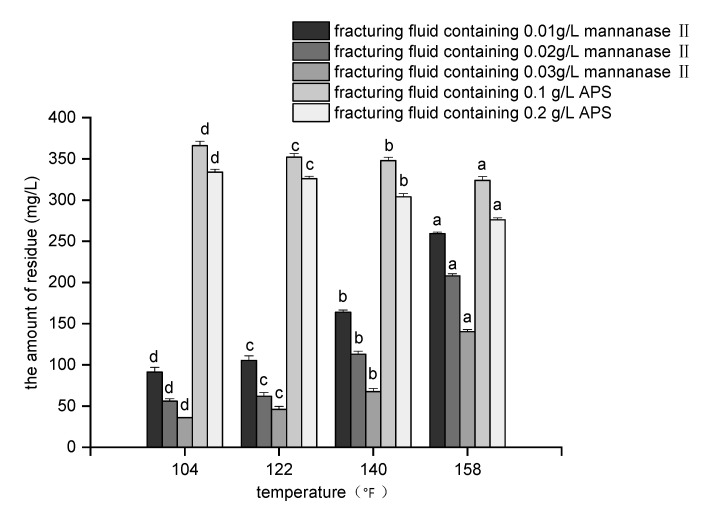
Residue-after-break results following the addition of different breakers under different temperature conditions (for each group of bars, the means indicated using different letters are significantly different at *p* < 0.05, where the vertical bars represent ± SD (*n* = 3)).

The effectiveness of the breaker can not only be characterized by viscosity of fracturing fluid, but also by the residual amount and particle size of polymer. The residual amount of polymer includes unbroken polymer and residues generated after the gel is broken. The presence of residue can cause flow reduction via the plugging of the formed fracture. Thus, a decrease in the amount of the residue corresponds to both decreased formation damage and increased production. In summary, the amount of residue is an important factor to consider in hydraulic fracturing. To evaluate the amount of residue generated by the breakers, residue-after-break experiments were conducted at 158 °F and other wider temperature range (Figure 4). The amount of residue generated by adding smaller concentrations of mannanase II was also <600 mg/L, which conforms to the criterion for evaluating the performance of water-based fracturing fluids [28,47]. Moreover, we observed that upon adding higher oxidizer concentrations (0.1 g/L) at the higher temperature of 158 °F, the mannanase II breaker still produced less residue than ammonium persulfate (Figure 4).

Figure 4 also demonstrates that, for a given amount of enzyme breaker, a higher temperature resulted in the generation of a greater amount of residue. In contrast, for a given amount of ammonium persulfate, a higher temperature resulted in the generation of a smaller amount of residue. This difference can be attributed to the different effects of temperature on the breakers [13,24,28].

**Figure 5:**
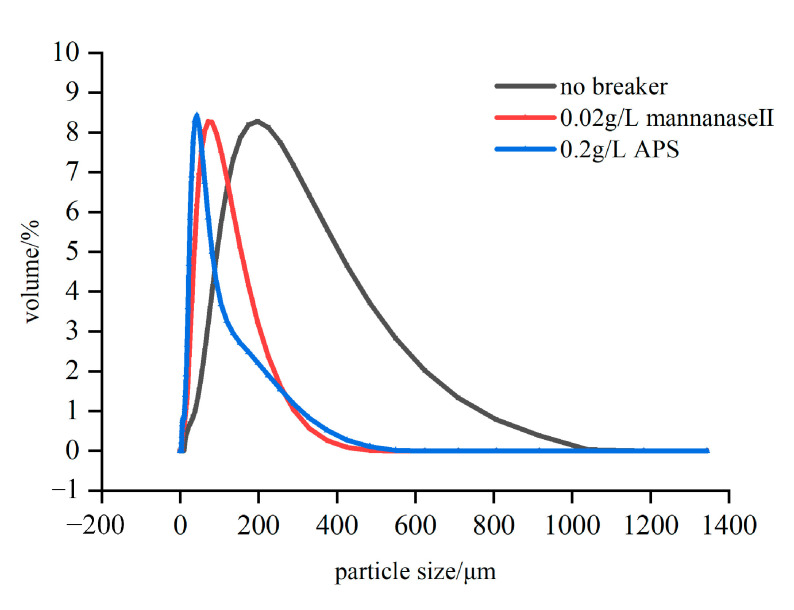
The change in the molecular size of the guar polymer during the gel-breaking process.

Compared with the oxidative breaker, the mannanase II induces more exhaustive and homogeneous breakage under the reservoir conditions [11,19]. To further verify this result, the experiment for the change in the molecular size of the guar during the gel-breaking process was carried out via a laser particle size analyzer at alkaline (pH = 8.5) and medium temperature (158 °F) conditions. Based on Figure 5, the molecular diameter of the fracturing fluid without the breaker is concentrated around 197.99 μm, while the molecular diameter after gel breaking with the mannanase II breaker is 42.7922 μm. The molecular diameter after gel breaking with a higher concentration of ammonium persulfate in the control group was 71.3062 μm. The experiment results showed that, compared with the use of ammonium persulfate breaker, there is a significant reduction in the size of the fracturing fluid after adding the enzyme breaker. Guar polymer molecules with this size can easily flow through the porous medium and improve reservoir conductivity [24].

Therefore, these results further confirm that mannanase II yields a better degradation performance than oxidative breakers. In addition, with its environmental benefits and cleaning ability, it can enhance both governmental and public trust in the hydraulic fracturing process.

### 3.4. Best-Use Concentrations of Mannanase II under Different Environmental Conditions

**Figure 6:**
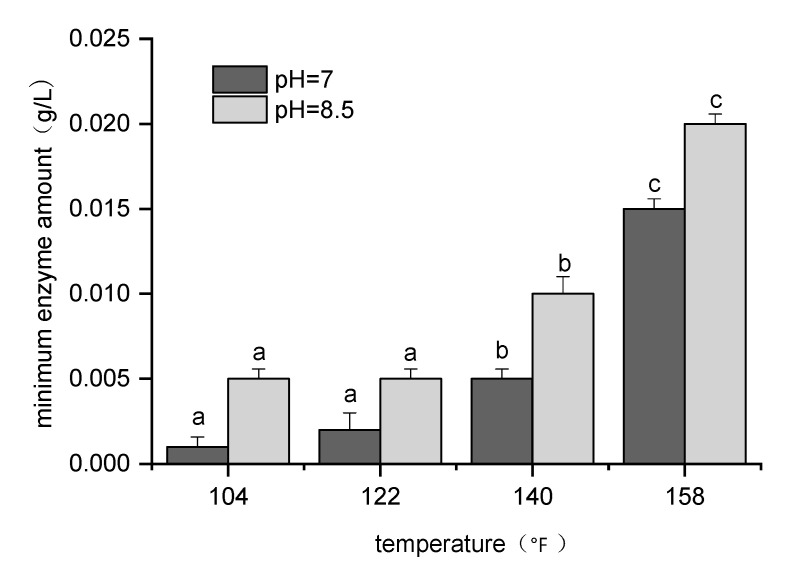
Plot of the minimum enzyme amount required to break the gel under different temperature and pH conditions (for each group of bars, the different letters indicate the means that are significantly different at *p* < 0.05, where the vertical bars represent ± SD (*n* = 3)).

The mannanase II breaker exhibited a dose–response effect. When the concentration of the enzyme breaker was too high, the fracturing fluid lost its shear resistance; however, when it was low, gel breaking was not complete [38,43]. The use of an appropriate amount of enzyme can ensure both environmental and economic benefits. Previous residue experiments have proven that enzyme breaker activity is affected by temperature. To establish guidelines for optimal mannanase II concentrations under different environmental conditions, histograms corresponding to the minimum enzyme amount required for gel breakage under different pH and temperature conditions were plotted (Figure 6). The results obtained demonstrate that any reduction in the gel viscosity depends on the breaker concentration, as well as on the temperature and pH of the fracturing fluid [6,22]. Thus, different concentrations of mannanase II at different temperatures and pH values showed different breakage results. Mannanase II exhibits highly efficient gel-breaking performance over a wide range of temperatures (104–158 °F) in both alkaline and neutral environments. Moreover, a temperature of 140 °F was identified as the critical point for the change in the activity of this breaker. The amount of enzyme required to break the gel increase significantly with an increase in the temperature. In addition, the enzymes showed higher activity in neutral environments [45,46]; therefore, the amount of mannanase II breaker required to break the gel in neutral environments was less than that required in alkaline environments.

## 4. Conclusions

In this study, we analyzed the feasibility of using the mannanase II enzyme in breaking borate-crosslinked guar gum gel under simulated the reservoir conditions (158 °F, pH = 8.5) of the Xinjiang Shengli Oilfield. The results obtained showed that, compared with other gel breakers, mannanase II exhibits superior gel-breaking performance. The residue measurement experiments showed that mannanase II provides a cleaner and more homogeneous polymer breakage than other breakers. Using a small quantity of mannanase II, it was possible to realize rapid gel-breaking while a much larger concentration of other enzymes was required to achieve similar results. The residue measurement experiments showed that mannanase II provides a cleaner and more homogeneous polymer breakage than other breakers. Experiments on the factors influencing the breaking performance also indirectly showed that mannanase II retains its activity over a wide range of application temperatures (104–158 °F) and pH values (7–8.5) in hydraulic fracturing. Overall, mannanase II was found to be a highly efficient, economical, and environmentally friendly breaker. Therefore, during oil production in low-permeability oil fields with medium temperatures and alkaline conditions, the advantages of mannanase II are unmatched by other gel breakers. Mannanase II is characterized by a better gel-breaking effect under low and medium temperature and neutral conditions. In future studies, we will conduct field experiments in low-permeability oil reservoirs to further demonstrate the excellent performance of this enzyme breaker. In addition, we speculate that this breaker has significant potential for applications in low-permeability oilfield development.

## Data Availability

Data is contained within the article.

## Sample Availability

Samples of the compounds are available from the authors.
